# Cord Blood 25-hydroxyvitamin D and Fetal Growth in the China-Anhui Birth Cohort Study

**DOI:** 10.1038/srep14930

**Published:** 2015-10-09

**Authors:** Peng Zhu, Shi-lu Tong, Wen-biao Hu, Jia-hu Hao, Rui-xue Tao, Kun Huang, Zhe Mou, Qi-fan Zhou, Xiao-min Jiang, Fang-biao Tao

**Affiliations:** 1 Department of Maternal, Child & Adolescent Health, Anhui Medical University, Hefei, China; 2 Anhui Provincial Key Laboratory of Population Health & Aristogenics, Anhui Medical University, Hefei, China; 3 School of Public Health and social work, and Institute of Health and Biomedical Innovation, Queensland University of Technology, Kelvin Grove, Qld, Australia; 4 Department of Gynecology and Obstetrics, Hefei First People’s Hospital, Hefei, China; 5 Shanghai Key Laboratory of Meteorology and Health, Shanghai, China; 6 Department of Gynecology and Obstetrics, Hefei Maternal and Child Health Hospital, Hefei, China

## Abstract

We determined the association of cord blood 25-hydroxyvitamin D [25(OH)D] with birth weight and the risk of small for gestational age (SGA). As part of the China-Anhui Birth Cohort (C-ABC) study, we measured cord blood levels of 25(OH)D in 1491 neonates in Hefei, China. The data on maternal sociodemographic characteristics, health status, lifestyle, birth outcomes were prospectively collected. Multiple regression models were used to estimate the association of 25(OH)D levels with birth weight and the risk of SGA. Compared with neonates in the lowest decile of cord blood 25(OH)D levels, neonates in four deciles (the fourth, fifth, sixth and seventh deciles) had significantly increased birth weight and decreased risk of SGA. Multiple linear regression models showed that per 10 nmol/L increase in cord blood 25(OH)D, birth weight increased by 61.0 g (95% CI: 31.9, 89.9) at concentrations less than 40 nmol/L, and then decreased by 68.5 g (95% CI: −110.5, −26.6) at concentrations from 40 to 70 nmol/L. This study provides the first epidemiological evidence that there was an inverted U shaped relationship between neonatal vitamin D status and fetal growth, and the risk of SGA reduced at moderate concentration.

Growth restriction in utero increases infant morbidity and mortality as well as the risk of adult chronic diseases^1^,^2^,^3^. The incidence of fetal growth restriction in both developed and developing countries progressively rose during the last two decades despite the introduction of many public health and medical interventions designed to reduce its occurrence^4^,^5^,^6^. Whether the common vitamin D deficiency during pregnancy contributes to any of the global adverse fetal growth epidemic remains unknown.

Maternal vitamin D deficiency during pregnancy is a significant public health problem globally^7^,^8^ due to the combined effects of limited sunlight exposure, inadequate intake, and additional growth needs of the fetus. The link between maternal vitamin D status and fetal growth, as measured most frequently by infant birth weight and small for gestational age (SGA), has been explored by a number of observational studies^9^,^10^,^11^,^12^,^13^,^14^,^15^ and randomized supplementation trials^16^,^17^,^18^,^19^,^20^ worldwide with mixed results.

Most studies assessed the associations of maternal 25-hydroxyvitamin D (25[OHD]) in early pregnancy with fetal growth. Evidences from some studies support the hypothesis that lower vitamin D status causes adverse fetal growth in a linear way, more severe deficiency would be expected to have a stronger effect than less severe deficiency or sufficiency^9^,^10^,^12^,^13^,^14^,^16^. However, some other studies don’t support this hypothesis^15^,^18^,^19^,^21^,^22^. The conflicting findings may be due to variations of study designs including the gestational weeks of vitamin D testing, the cut-point of vitamin D, ethnicity of the study population, genetic variation and outcome variables. The translation of these findings to clinical practice has not occurred.

Notably, increased risks for a range of health outcomes, including SGA, among pregnant women with high 25(OH)D levels has been reported by several studies^11^,^23^. Although fetal and newborn concentrations of 25(OH)D depend on and correlate with maternal serum levels^18^,^24^, there has been a paucity of data about the association between cord blood 25(OH)D concentration and birth outcomes. In this study, we examined cord blood levels of 25(OH)D in 1491 neonates in Hefei, China and assessed the association of cord blood levels of 25(OH)D with birth weight and the risk of SGA.

## Results

Attrition analyses showed the distributions of sociodemographic characteristics, health status, lifestyle and birth outcomes, non-participants did not differ from participants. In this study, the mean age of the participants was 27.65 years (SD = 3.66), the majority (79.2%) of participants had completed high school and beyond (>9 years), and the income of 84.9% of the participants was more than 2 000 RMB per month. The mean gestational age at delivery was 38.91 weeks (SD = 1.47), ranging from 32 to 42 weeks, with 96 preterm births (5.3%). The mean birth weight was 3383.1 g (SD = 453.9), ranging from 1500 to 5500 g, with 133 small for gestational age infants (8.9%). In our sample, 791 (53.1%) newborns were males. The mean cord blood 25(OH)D level was 39.43 nmol/L (SD = 20.35), ranged from 6.06 to 119.64. Table 1 shows the characteristics of 1 491 pregnant women across increasing deciles of cord blood 25-hydoxyvitamin D levles. With increasing deciles of cord blood 25(OH)D, the percentage of birth during summer to autumn and male infant increased (*P* < 0.05 for the trend). Male infants had slightly higher cord blood 25(OH)D levels than females, but the difference did not reach statistical significance after adjusting for the season of birth (*P* = 0.11).

Table 2 shows the associations between socio-demographic characteristics, lifestyle variables, birth related variables and baby’s birth weight. Factors that were significantly associated with an increase in birth weight were: being older, having a better education (>9 years vs ≤9 years), having at least one child (multipara vs primipara), being male, and having a longer gestation.

In a multiple linear regression model, the deciles of cord blood 25(OH)D were significantly associated with birth weight, even after adjustment for gestational week, infant gender, prepregnancy BMI, gestational weight gain (GWG), birth season, maternal age, education, income, alcohol consumption, pregnancy complications, parity, paternal smoking and alcohol consumption. Neonates in four deciles (the fourth, fifth, sixth and seventh deciles) had significantly increased birth weight compared with those in the lowest decile. The highest adjusted regression coefficients was observed in the fifth decile (β:176.5; 95% CI: 86.4, 266.5) (Table 3).

**Figure 1:**
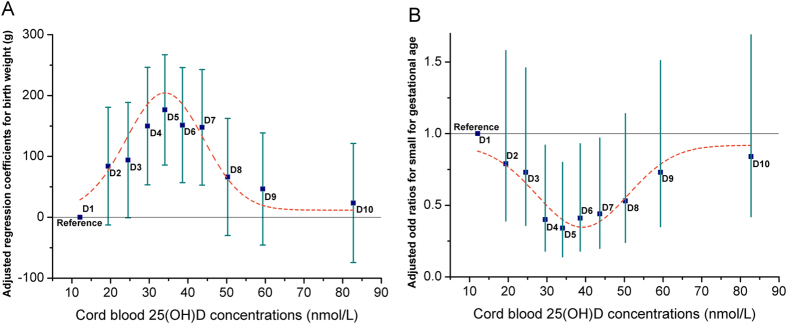
Fully adjusted regression coefficients (A) for birth weight and odd ratios (B) of small for gestational age across the deciles of cord blood 25(OH)D concentrations. Adjusted gestational week, infant gender, prepregnancy BMI, GWG, birth season, maternal age, education, income, alcohol consumption, pregnancy complications, parity, paternal smoking and alcohol consumption. D1, D2 and D3 means the first, second and third decile of cord blood 25(OH)D respectively, and so on. The dotted line represents the trend for the point estimate.

Consistently, in a multiple logistic regression model with full adjustment described above (except gestational weeks), the deciles of cord blood 25(OH)D were significantly associated with risk of SGA. Neonates in four deciles (the fourth, fifth, sixth and seventh deciles) had significantly decreased risk of SGA compared with those in the lowest decile, with the lowest adjusted OR observed in the fifth decile [0.34 (95% CI 0.14–0.80)] (Table 3). Figure 1 depicts these joint nonlinear associations.

**Figure 2:**
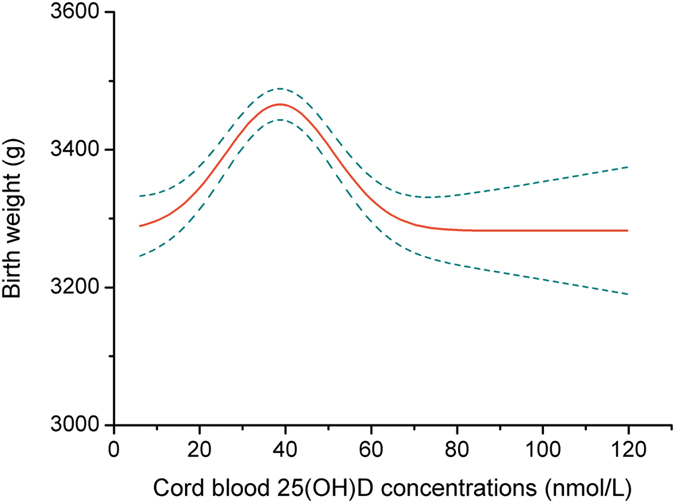
Unadjusted association of cord blood 25(OH)D concentrations and birth weight using Gaussian curve-fitting model with knot at 40 nmol/L (*P* < 0.001). The solid line represents the trend for changes in birth weight in mean values across increasing cord blood 25(OH)D concentrations and dotted lines represent the 95% confidence interval.

We further assessed the dose-response relation between cord blood 25(OH)D levels and birth weight. The inverted U-shaped nature of the association of cord blood 25(OH) D with birth weight was confirmed with Gaussian curve-fitting model (*P* < 0.001). Indeed, this model suggested a significant steep increase in birth weight up to 40 nmol/L of cord blood 25(OH)D and a gradual downward trend in birth weight at concentrations from 40 to 70 nmol/L, and then level off thereafter (Fig. 2).

**Figure 3:**
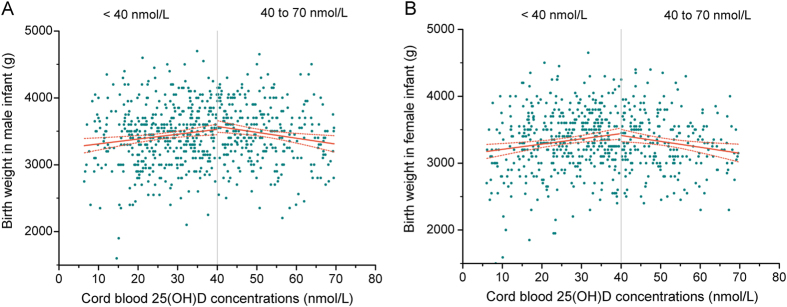
Adjusted linear regression associations between cord blood 25(OH)D concentrations and birth weight at 25(OH)D < 40 nmol/L and from 40 to 70 nmol/L according to infant gender. The solid line represents the linear regression trend for the change of birth weight in mean values across increasing cord blood 25(OH)D concentrations.

Multiple linear regression model shows that per 10 nmol/L increase in cord blood 25(OH)D levels, birth weight increased by 61.0 g (95% CI: 31.9, 89.9) at concentrations less than 40 nmol/L, but decreased by 68.5 g (95% CI: −110.5, −26.6) at concentrations from 40 to 70 nmol/L. We further assessed the difference of association between cord blood 25(OH)D and birth weight in male and female infants. After adjustment for confounders, average birth weight of boys and girls increased by 62.6 g (95% CI: 21.2, 104.0) and 68.0 g (95% CI: 27.1, 109.0), respectively, per 10 nmol/L increase in cord blood 25(OH)D up to 40 nmol/L. However, average birth weight of boys and girls decreased by 58.7 g (95% CI: −115.9, −1.5) and 82.3 g (95% CI: −146.5, −18.1), respectively, per 10 nmol/L increase in cord blood 25(OH)D from 40 to 70 nmol/L (Fig. 3).

## Discussion

To our knowledge, this is a study with the largest sample examining the association of cord blood 25(OH)D concentrations with birth weight and the risk of fetal growth restriction. The estimated difference in mean of birth weight among neonates in the fifth decile compared with those in the lowest decile was around 176 g, greater than the reduction in birth weight observed for children exposed to maternal smoking in utero^25^. Thus, the potential public health implications of our findings are substantial.

Most previous studies in this field were undertaken in developed countries^13^,^14^,^15^. Compared with North American and European countries (the mean 25(OH)D levels of pregnant women in US and UK is approximately 50 nmol/L), Asian countries maybe have more pregnant women with vitamin D deficiency^13^,^26^,^27^,^28^,^29^. For example, the average 25(OH)D level of pregnant women in Beijing (39 °N) was only 28.6 nmol/L^26^, which was even lower than the average 25(OH)D levels (39.4 nmol/L) of newborns in Hefei (32 °N) reported in our study. However, it should be underlined that the difference on 25(OH)D levels may be partly attributed to the different season of blood drawn and assay methods.

Currently, there is limited information on the association between fetal vitamin D status and fetal growth. A small cross-sectional study in China demonstrated that average birth weight was lower in newborns (n = 27) with 25(OH)D < 25 nmol/L than those (n = 31) with 25(OH)D ≥25 nmol/L^26^. An analysis of 734 neonates in US^13^ indicated that lower neonatal 25(OH)D were associated with lower birth weight-for-gestational age and higher odds of SGA. Another case-control study suggested that infants born before 32 wks’ gestation had an increased risk of vitamin D deficiency compared with mature infant, however, there was no statistically significant difference in mean cord blood 25(OH)D levels between SGA and non-SGA infants^29^.

Previous studies have several limitations including small sample sizes, inconsistent definitions for fetal vitamin D status, few adjustment for confounders and only linear models performed. In this study with a large sample, adjustment for more confounders and using non-linear models, we found that there was an inverted U-shaped relationship between cord blood 25(OH)D and birth weight, suggesting that the newborn with low or high levels of fetal vitamin D are both in increased risk of growth restriction and higher levels of fetal vitamin D have not been shown to confer greater benefits. However, it should be emphasized that the inverted U shaped relationship in this study does not imply causality, because fetal weight would have to have been affected by 25(OH)D at critical times of development rather than at birth when samples were collected. Additionally, although there was an inverted U shaped relationship, the infants above the seventh decile of 25(OH)D levels still had higher birth weight than those in the first decile.

The finding that vitamin D levels in the upper range increased the health risk in this study was consistent with previous studies examining the risk of fetal growth restriction^11^, allergic response^30^,^31^, lower-extremity function^32^, frailty status^33^, as well as cardiovascular disease^34^,^35^. Although the neonatal 25(OH)D levels at birth cannot fully represent the vitamin D status during critical times of gestation due to a half-life of several weeks of 25(OH)D^34^, it was quite possible that the neonates with lower or higher cord blood 25(OH)D levels may also have lower or higher average levels of 25(OH)D during the whole pregnancy. Therefore, the inverted U shaped relationship in this study maybe suggested that gestational vitamin D may have dual biological effects on the fetal growth. However, the biological mechanisms remain uncertain. Studies in adults have indicated that there was a U-shaped association between 25(OH)D and C-reactive protein (CRP), and increasing 25(OH)D concentrations may also be related to proinflammatory states^36^,^37^. We speculate that fetal exposure to elevated CRP associated with 25(OH)D in lower or higher range may result in fetal growth restriction^38^,^39^, which might explain the inverted U-shaped relationship between 25(OH)D levels and fetal growth in this study. However, we cannot rule out residual confounding as a potential explanation, especially whether genotypic differences in the study population may explain this inverted U-shaped association. Further research is needed to understand the mechanisms.

Our study has three major strengths. First, it is the first study reporting that there was an inverted U-shaped relationship between cord blood 25(OH)D and fetal growth. Second, prospective data collection procedure, a relatively large sample size and statistical adjustment for a large number of confounders were used. Third, utility of both continuous measure and the deciles of cord blood 25(OH)D concentrations helped to detect the inverted U-shaped relation.

However, several limitations in this study should also be acknowledged. First, we only measured 25(OH)D concentrations, which is one member of the vitamin D family. At birth, there are potential changes to levels of vitamin D binding protein, 1,25(OH)2D, the CYP27 enzyme and expression of vitamin D receptors^40^, which may influence the association between 25(OH)D and fetal growth^41^. Thus, future studies of vitamin D and growth restriction would benefit from the consideration of other vitamin D family members. Second, 25(OH)D levels at birth cannot represent the vitamin D status in early pregnancy. However, we were unable to assess the effect of vitamin D levels during early pregnancy on fetal growth as these data are unavailable. Third, the absence of data on outdoor activity, dietary intake, environmental factors and genetic variation may result in residual confounding in this study. Finally, this study was conducted in one city and caution is necessary in generalizing the findings to other regions due to the strong influence of latitude.

In conclusion, this study provides the first epidemiological evidence that there was an inverted U shaped relationship between neonatal vitamin D status and fetal growth. The findings suggested that vitamin D supplements during pregnancy should be encouraged, but with caution. If confirmed in further studies, it could have important implications for public health policy.

## Methods

### Study subjects

This study was conducted on the basis of a prospective birth cohort comprising 2552 pregnant women recruited in Hefei (32 °N latitude) from January to September 2008. As part of the C-ABC study^42^, pregnant women who received prenatal check-ups in Hefei Maternal and Child Health Hospital were recruited by a team of midwives, nurse and health professionals. Participants completed a structured questionnaire including sociodemographic characteristics and lifestyle. At birth, midwives or study nurses collected the newborn’s anthropometric details and cord blood, when available. In this study, stillbirth (n = 11), birth defect (n = 12), women with delivery before 32 weeks of gestation (n = 14), pregnancy with assisted reproductive technology (n = 6), or multiple gestations (n = 48) were excluded from the study. Finally, we obtained full data including cord blood from 1491 mother-infant pairs. The study was approved by the Ethics Committee of the Anhui Medical University. All experiments in this study were performed in accordance with the relevant guidelines and regulations and written informed consent was obtained from each participants.

### Cord blood 25-hydoxyvitamin D

Cord blood sample was collected immediately after delivery and anticoagulated by use of sodium heparin. Plasma samples were centrifuged and promptly refrigerated at −4 °C, within 12 h, transferred to −80 °C freezers for long-term storage. 25(OH)D concentrations were measured using the commercial radioimmunoassay kits (DiaSorin Stillwater, MN, USA). Intra-assay and inter-assay coefficients of variation were 8.8% and 11.1% respectively. Plasma concentrations of 25(OH)D were analyzed both as a continuous variable and a decile.

### Outcomes variables

The key outcomes assessed in this study included birth weight and SGA defined as birth weight <10th percentiles of distribution for gestational age and infant gender^43^. The accuracy of scales used to measure birth weight in the hospital was checked using standard weights at the beginning of the study and every 3 months thereafter.

### Potential confounders

The most important potential confounder was gestational age, infants gender, prepregnancy body mass index (BMI), GWG and birth season, which have been known to be associated with birth weight or maternal 25(OH)D levels^15^,^44^. The gestational age (in completed weeks) based on the difference between the date of the last menstrual period and the date of delivery and was categorized as <37 or ≥37 gestational weeks. Prepregnancy BMI was calculated on the basis of the height routinely measured at the clinic visit and on the self-reported prepregnancy weight obtained at interview and were categorized as underweight (<18.5), normal weight (18.5–23.9), or overweight or obese (≥24.0)^45^. The absolute amount of weight gained throughout pregnancy was determined by subtracting the self-reported prepregnancy weight at interview from the measured weight recorded at the last prenatal visit before delivery, categorized based on quartiles. Birth season was designated as: winter (December, January, February), spring (March, April, May), summer (June, July, August), or fall (September, October, November). Other potential confounders included maternal age (20–24, 25–29 and 30 and more years), education (≤9 and >9 years of completed schooling), household income (less than 2000, 2000–4000 and more than 4000 RMB), parity (primipara or multipara), pregnancy complications, maternal alcohol consumption during pregnancy (any or no), paternal alcohol consumption (any or no) and smoking (none, 1–5, 6 or more cigarettes daily) during pregnancy.

### Statistical analysis

Cord blood 25(OH)D concentrations were divided into deciles. General linear regression models and Mantel-Haenszel chi-square test were used to compare means and proportions for the characteristics of pregnancy women across the deciles of 25(OH)D level. The means of the deciles were fit as continuous variables to estimate the trend of variables across deciles in a linear regression model. Differences of birth weight according to maternal characteristics were evaluated using linear regression models.

The adjusted regression coefficients of multiple linear regression models were generated for the association between the deciles of 25(OH)D levels and birth weight, that were adjusted for gestational week, infant gender, prepregnancy BMI, GWG and birth season, and further adjusted for maternal age, education, income, alcohol consumption, pregnancy complications, parity, paternal smoking and alcohol consumption. Multivariable logistic regression models were used to determine odd ratios (OR) and 95% CIs for SGA after adjusting for confounders as described above.

The curvilinear association between 25(OH)D and birth weight was best described using a Gaussian curve-fitting model^46^ with knot at 40 nmol/L. Then, adjusted regression coefficients of linear regression model were generated for the association between 25(OH)D and birth weight according to 25(OH)D level with knot at 40 nmol/L. Furthermore, the data were stratified by infant gender. All statistical analyses were performed using SPSS statistical software, version 21.0.

## Additional Information

**How to cite this article**: Zhu, P. *et al.* Cord Blood 25-hydroxyvitamin D and Fetal Growth in the China-Anhui Birth Cohort Study. *Sci. Rep.* **5**, 14930; doi: 10.1038/srep14930 (2015).

## Figures and Tables

**Table 1:** Characteristics of the study population across percentile increments of cord blood 25(OH)D levels.

Characteristics	Percentile of 25(OH)D level in cord blood										*P*-values*
	1–10 (n = 147)	11–20 (n = 151)	21–30 (n = 149)	31–40 (n = 149)	41–50 (n = 149)	51–60 (n = 149)	61–70 (n = 150)	71–80 (n = 148)	81–90 (n = 150)	91–100 (n = 149)	
Cord blood 25(OH) , nmol/L)											
Mean (SD)	12.08 (3.07)	19.37 (1.66)	24.50 (1.45)	29.54 (1.43)	34.05 (1.09)	38.58 (1.43)	43.66 (1.58)	50.25 (2.11)	59.34 (3.76)	82.78 (13.99)	
Range	6.06–16.59	16.62–22.17	22.20–27.15	27.18–32.01	32.07–36.03	36.06–41.07	41.10–46.71	46.83–53.85	53.91–66.72	66.78–119.64	
Sociodemographic characteristics											
Maternal age, y, mean (SD)	27.00 (3.42)	27.72 (3.68)	27.42 (3.51)	28.15 (3.96)	27.74 (3.51)	27.56 (3.42)	27.63 (3.74)	27.93 (3.78)	28.01 (4.02)	27.34 (3.44)	0.067
Maternal education <9 years, *n* (%)	33(22.4)	34(22.5)	33(22.1)	36(24.2)	29(19.5)	24(16.1)	25(16.7)	33(22.3)	29(19.3)	34(22.8)	0.209
Family monthly income <2000 RMB/yuan, *n* (%)	27(18.4)	25(16.6)	17(11.4)	21(14.1)	22(14.8)	19(12.8)	25(16.7)	30(20.3)	17(11.3)	22(14.8)	0.613
Perinatal health status											
Prepregnancy BMI, mean (SD)	20.20 (2.73)	20.25 (2.27)	20.37 (2.64)	20.10 (2.13)	19.80 (2.36)	20.03 (2.22)	20.04 (2.53)	20.55 (2.57)	20.27 (2.51)	19.97 (2.08)	0.547
GWG, kg, mean (SD)	16.68 (4.63)	17.19 (5.31)	17.43 (5.19)	16.74 (4.47)	16.93 (5.39)	17.02 (5.07)	16.79 (4.59)	17.01 (4.32)	16.24 (4.58)	15.53 (4.73)	0.150
Multipara, *n* (%)	15(10.2)	26(17.2)	14(9.4)	22(14.8)	22(14.8)	24(16.1)	22(14.7)	16(10.8)	22(14.7)	133(10.7)	0.637
Pregnancy complications^a^, *n* (%)	23(15.6)	33(21.9)	22(14.8)	28(18.8)	23(15.4)	22(14.8)	18(12.0)	16(10.8)	23(15.3)	18(12.1)	0.055
Prepregnancy lifestyle^b^											
Maternal alcohol consumption^c^, *n* (%)	18(12.2)	30(19.9)	17(11.4)	25(16.8)	20(13.4)	25(16.8)	29(19.3)	24(16.2)	22(14.7)	15(10.4)	0.537
Paternal smoking^d^, *n* (%)	35(23.8)	29(19.2)	38(25.5)	35(23.5)	27(18.1)	40(26.8)	29(19.3)	35(23.6)	29(19.3)	39(26.2)	0.640
Paternal alcohol consumption^c^, *n* (%)	114(77.6)	117(77.5)	127(85.2)	116(77.9)	111(74.5)	120(80.5)	117(78.0)	126(85.1)	127(84.7)	124(83.2)	0.118
Birth outcomes											
Male infant, *n* (%)	62(42.2)	86(57.0)	73(49.0)	80(53.7)	76(51.0)	78(52.3)	85(56.7)	81(54.7)	87(58.0)	83(55.7)	0.027
Gestational weeks, w, mean (SD)	38.77 (1.98)	38.68 (1.47)	39.04 (1.37)	39.00 (1.22)	38.93 (1.57)	38.98 (1.41)	38.96 (1.28)	39.13 (1.40)	38.89 (1.29)	38.77 (1.60)	0.204
Birth weight, g, mean (SD)	3235.6 (533.4)	3351.4 (455.4)	3382.3 (452.2)	3447.6 (412.3)	3460.1 (435.6)	3452.3 (475.6)	3436.5 (416.0)	3412.8 (437.4)	3342.4 (441.1)	3310.4 (425.7)	0.262
SGA, *n* (%)	22 (15.0)	17 (11.3)	16 (10.7)	9 (6.0)	8 (5.4)	9 (6.0)	10 (6.7)	11 (7.4)	14 (9.3)	17 (11.4)	0.155
Birth during summer or autumn, *n* (%)	0 (0.0)	17 (11.3)	45 (30.2)	66 (44.3)	85 (57.0)	90 (60.4)	105 (70.0)	116 (78.4)	128 (85.3)	140 (94.0)	<0.001

Abbreviation: 25(OH)D, 25-hydroxyvitamin D; BMI, body mass index; GWG, gestational weight gain; SGA, small for gestational age.

^a^Pregnancy complications included diabetes mellitus, hypertension, abnormal heart function, glandula thyreoidea disease, intrahepatic cholestasis of pregnancy, moderate and severe anemia.

^b^Prepregnancy lifestyle means lifestyle during up to 6 months before pregnancy.

^c^Alcohol consumption was defined as any alcohol consumption.

^d^Paternal smoking was defined as more than 6 cigarettes daily.

^*^Test for trend based on Mantel-Haenszel chi-square test for categorical variables and linear regression for continuous variables.

**Table 2:** Differences in birth weight according to sociodemographic, health status, lifestyle and birth outcomes.

Characteristics	*n* (% 1491)	β (95%CI)	*P* value
Sociodemographic characteristics			
Maternal age, y			
20–24	252 (16.9)	Reference	
25–29	870 (58.4)	130.2 (67.0, 193.5)	<0.001
≥30	369 (24.7)	146.4 (70.1, 222.8)	<0.001
Maternal education, education year			
≤9	310 (20.8)	Reference	
>9	1181 (79.2)	87.5 (30.8, 144.2)	0.002
Family monthly income, yuan/RMB			
<2000	225 (15.1)	Reference	
2000–4000	1100 (73.8)	46.2 (−18.9, 111.3)	0.164
>4000	166 (11.1)	104.7 (9.6, 199.8)	0.031
Perinatal health status			
Prepregnancy BMI			
Underweight (<18.5)	362 (24.3)	−142.1 (−195.4, −88.9)	<0.001
Normal (18.5–23.9)	1036 (69.5)	Reference	
Overweight or obesity (≥24.0)	93 (6.2)	184.0 (86.1, 281.8)	<0.001
GWG			
Quartile 1(lowest)	367 (24.6)	Reference	
Quartile 2	303 (20.3)	73.5 (3.2, 143.8)	0.040
Quartile 3	423 (28.4)	217.6 (155.9, 279.4)	<0.001
Quartile 4(highest)	398 (26.7)	279.9 (217.6, 342.2)	<0.001
Parity			
Primipara	1292 (86.7)	Reference	
Multipara	199 (13.3)	76.8 (9.7, 143.8)	0.025
Pregnancy complications^a^			
None	1265 (84.8)	Reference	
Yes	226 (15.2)	−3.9 (−68.2, 60.4)	0.906
Prepregnancy lifestyle^b^			
Maternal alcohol consumption			
None	1266 (84.9)	Reference	
Any	225 (15.1)	22.7 (−41.7, 87.2)	0.489
Paternal smoking			
None	849 (57.0)	Reference	
1–5 cigarettes daily	306 (20.5)	−13.5 (−71.2, 44.2)	0.646
≥6 cigarettes daily	336 (22.5)	−39.4 (−97.2, 18.4)	0.181
Paternal alcohol consumption			
None	292 (19.6)	Reference	
Any	1199 (80.4)	41.2 (−16.9, 99.2)	0.165
Birth outcomes			
Infant gender			
Male	791 (53.1)	Reference	
Female	700 (46.9)	−129.6 (−175.3, −83.8)	<0.001
Gestational weeks			
Full-term (≥37 weeks)	1419 (95.2)	Reference	
Premature (<37 weeks)	72 (4.8)	−767.7 (−860.0, −667.4)	<0.001
Birth season			
Summer- Autumn	792 (53.1)	Reference	
Winter- Spring	699 (46.9)	−5.3 (−51.6, 40.9)	0.820

Abbreviation: BMI, body mass index; GWG, gestational weight gain.

^a^Pregnancy complications included diabetes mellitus, hypertension, abnormal heart function, glandula thyreoidea disease, intrahepatic cholestasis of pregnancy, moderate and severe anemia.

^b^Prepregnancy lifestyle means lifestyle during up to 6 months before pregnancy.

**Table 3:** Associations of cord blood 25(OH)D levels with birth weight and the risk of SGA.

			Birth Weight				SGA			
Cord Blood 25(OH)D Levels (nmol/L)		*n*	Partially Adjusted^a^		Fully Adjusted^b^		Partially Adjusted^a^		Fully Adjusted^b^	
Percentile	Mean (SD)		β (95% CI)	*P*-values	β (95% CI)	*P*-values	OR (95% CI)	*P*-values	OR (95% CI)	*P*-values
1–10	12.08 (3.07)	147	Reference		Reference		Reference		Reference	
11–20	19.37 (1.66)	151	96.1 (−0.8, 192.9)	0.052	84.1 (−12.7, 181.0)	0.089	0.77 (0.39, 1.52)	0.452	0.79 (0.39, 1.58)	0.500
21–30	24.50 (1.45)	149	108.5 (14.5, 202.4)	0.024	94.4 (−0.1, 189.2)	0.050	0.71 (0.35, 1.41)	0.324	0.73 (0.36, 1.46)	0.371
31–40	29.54 (1.43)	149	170.6 (76.0, 265.1)	<0.001	150.0 (53.0, 246.1)	0.002	0.38 (0.17, 0.86)	0.021	0.40 (0.18, 0.92)	0.030
41–50	34.05 (1.09)	149	189.1 (99.8, 278.4)	<0.001	176.5 (86.4, 266.5)	<0.001	0.33 (0.14, 0.77)	0.010	0.34 (0.14, 0.80)	0.013
51–60	38.58 (1.43)	149	161.6 (67.9, 255.3)	0.001	151.4 (56.9, 245.9)	0.002	0.38 (0.17, 0.86)	0.020	0.41 (0.18, 0.93)	0.033
61–70	43.66 (1.58)	150	161.2 (67.7, 254.7)	0.001	147.8 (52.8, 242.7)	0.002	0.42 (0.19, 0.93)	0.032	0.44 (0.20, 0.97)	0.042
71–80	50.25 (2.11)	148	92.2 (−4.7, 189.1)	0.062	66.2 (−30.0, 162.5)	0.177	0.49 (0.23, 1.06)	0.072	0.53 (0.24, 1.14)	0.105
81–90	59.34 (3.76)	150	56.9 (−34.4, 148.2)	0.221	46.5 (−45.6, 138.6)	0.321	0.63 (0.31, 1.28)	0.201	0.73 (0.35, 1.51)	0.400
91–100	82.78 (13.99)	149	49.6 (−46.9, 146.1)	0.313	23.5 (−74.4, 121.4)	0.637	0.78 (0.40, 1.55)	0.485	0.84 (0.42, 1.69)	0.631

Abbreviation: 25(OH)D, 25-hydroxyvitamin D; BMI, body mass index; GWG, gestational weight gain; SGA, small for gestational age.

^a^Adjusted for gestational week, infant gender, prepregnancy BMI, GWG and birth season.

^b^Further adjusted for maternal age, education, income, alcohol consumption, pregnancy complications, parity, paternal smoking and alcohol consumption additionally.
